# The Effect of Probiotics on the Management of Pain and Inflammation in Osteoarthritis: A Systematic Review and Meta-Analysis of Clinical Studies

**DOI:** 10.3390/nu16142243

**Published:** 2024-07-12

**Authors:** Maria Moyseos, Jenny Michael, Nuno Ferreira, Antonia Sophocleous

**Affiliations:** 1 Department of Life Sciences, School of Sciences, European University of Cyprus, 6, Diogenes Str., Nicosia 2404, Cyprus; maria.moyseos@cyric.eu (M.M.); tzen.ny1998@hotmail.com (J.M.); 2 Cyprus Research & Innovation Centre (CYRIC), 72, 28th October Avenue, Nicosia 2414, Cyprus; 3 Department of Social Sciences, University of Nicosia, 46, Makedonitissas Avenue, Nicosia 2417, Cyprus; ferreira.n@unic.ac.cy

**Keywords:** osteoarthritis, articular cartilage damage, pain, inflammation, probiotics, systematic review, meta-analysis

## Abstract

Osteoarthritis (OA) is one of the most common musculoskeletal disorders. Recently, research has focused on the role of intestinal microbiome dysbiosis in OA. The aim of this study was to systematically review randomized intervention clinical studies investigating the effect of probiotics on the management of OA-related pain and inflammation. Pre-clinical studies and non-randomized trials were excluded. A literature search was conducted using MEDLINE, EMBASE, and Web of Science. Study quality was assessed with the Cochrane risk of bias (RoB2) tool and the Risk of Bias in N-of-1 Trials (RoBiNT) scale. RevMan was used for the meta-analysis. Outcome measures assessed self-reported pain, stiffness and impediment, and serum hs-CRP. Three studies, with 501 participants, were considered eligible for qualitative synthesis and meta-analysis. A significant reduction in symptoms across all outcomes measured, except stiffness, was evident with *Lactobacillus casei* Shirota. However, all other probiotics reviewed did not seem to have any effect on the measured outcomes. Pre-clinical evidence, along with the RCTs reviewed, suggests that probiotics of the *Lactobacillus* strains might be of use for managing pain and inflammation in OA. Considering the small number of studies included in the present review and the possible risk of bias, we conclude that further studies on the role of probiotics in humans with OA are warranted.

## 1. Introduction

Osteoarthritis (OA) is the most common form of arthritis, affecting half a billion people in the world (in 2021) and expected to affect up to 1 billion people by 2050 [1]. OA is a chronic degenerative disease characterized by a loss or erosion of articular cartilage, defects in the subchondral bone, the presence of osteophytes, and mild inflammation of the synovial membrane, causing pain, stiffness, and mobility difficulties primarily in the knee, hip, and hand joints [2]. Primary risk factors for the development of OA include age and metabolic syndrome, which presents as insulin resistance, obesity, vascular pathology, and dyslipidemia, but also through low-grade systemic inflammation [3,4]. This chronic inflammatory stress is linked to the release of pro-inflammatory OA pathogenesis-related cytokines including tumor necrosis factor (TNF)-alpha, matrix metalloproteinases (MMPs), interleukin (IL)-1, IL-2, IL-6, IL-7, IL-15, and IL-21 [5]. This pattern of chronic inflammation has been similarly observed in gut microbiota dysbiosis, hence positing the question of whether the “gut–joint axis” is part of the multifactorial causal nature of OA [6]. In fact, a review by several experts in the field has conclusively established a link between gut microbiome dysbiosis and OA [7]. The putative mechanism of this link is that, through genetics, dietary changes, and certain medication uses, a disruption in the normal functioning of the gut is caused, which leads to this chronic inflammation process that ultimately presents as OA (for a comprehensive review see [6]).

With no existing cure, most treatment options focus on the management of the key symptom, of pain. Current guidelines suggest the use of acetaminophen, nonsteroidal anti-inflammatory drugs (NSAIDs), and intraarticular glucocorticoid injections as a way of reducing pain and the inflammation of the affected joint [8,9,10]. However, the long-term use of these treatment approaches can be risky as it might interfere with patient co-morbidities and increase the risk of adverse events occurring [11]. Further to this, several studies point to the use of these pain management strategies as potential sources of gut microbiome dysbiosis [12,13,14]. Therefore, there has been an increase in interest in alternative forms of treatment that have fewer secondary effects or gut dysbiosis involvement. One potential alternative that has been identified is that of dietary supplements. In fact, many people with OA (35–69% [15,16]) already regularly use numerous dietary supplements (e.g., collagen, L-carnitine, Glucosamine) to target their OA symptoms [17]. However, current evidence is still in its infancy, and studies are lacking in quality and quantity for any clear recommendations to be made about dietary supplements, with a recent review showing no significant benefits of supplement use in the mid- to long-term management of pain and function in OA [17]. One new avenue of research linked to dietary supplements that has not been scrutinized is that of the use of probiotics for OA.

The Food and Agriculture Organization (FAO) and the World Health Organization (WHO) define probiotics as “live microorganisms that, when administered in sufficient amounts, confer a health benefit to the host” [18]. Probiotics are commonly deployed in the host’s intestine and, through interactions with the host cells, cause changes in the composition of the intestinal flora, impacting metabolism and immunity, with beneficial effects [18]. Probiotic supplementation used in human nutrition stems primarily from lactic acid bacteria such as *Lactobacillus* (such as *Lactobacillus casei*, *Lactobacillus rhamnosus*, *Lactobacillus plantarum*, *Lactobacillus helveticus*), *Bifidobacterium* (such as *Bifidobacterium breve*, *Bifidobacterium longum*, *Bifidobacterium infantis*), or yeasts such as *Saccharomyces boulardii* [19,20].

Several preclinical and clinical studies have established the potential of the use of probiotics in the management of OA [6]. For example, a recent mice model study showed that the reconstitution of the microbiome in association with probiotic supplementation conferred a protective effect against cartilage damage related to osteoarthritis in the medial femoral condyle compartment of the joint. Further to these effects, changes were noted in subchondral bones, particularly the femoral condyle, where trabecular bone volume, trabecular thickness, and subchondral plate thickness were observed. Concurrently, changes in the tibial compartment were also noted, although they were less pronounced than those seen in the distal femur and were most evident in the un-operated knee [21]. In human trials, probiotics also seem to demonstrate a capacity to alleviate symptoms of OA). Lei and colleagues [22] reported that *Lactobacillus casei* Shirota (LcS) has the potential to reduce inflammatory joint damage caused by OA in the knee when compared to a placebo. Likewise, Lyu et al. [23] showed that *S. thermophilus* (TCI633), a recently recognized strain of probiotics from human breast milk, can improve knee OA degeneration. The latter was also supported by the experimental findings of an anterior cruciate ligament transection (ACLT)-induced OA rat model, which included improvements in knee joint swelling, joint tissue inflammation, and cartilage damage [23].

Therefore, this article aims to review the existing evidence on the effect of the use of probiotics in the management of OA-related pain and inflammation.

## 2. Materials and Methods

The Preferred Reporting Items for Systematic Review and Meta-Analysis (PRISMA) criteria were used for conducting and reporting the results of this systematic review [24]. For the PRISAM 2020 Checklist and the PRISMA 2020 for Abstract Checklist, please see Table S4 and Table S5, respectively.

### 2.1. Search Strategy

The following three databases were used to perform the systematic search on 9th January 2024: Medline [Ovid MEDLINE(R) ALL <1946 to 8 January 2024>], EMBASE (Embase Classic + Embase <1947 to 2024 Week 01>), and Web of Science (WOS: 1900 to 2024). Medical subject heading (MeSH) keywords and free words, along with their synonyms, were used to search each database for the concepts “probiotics”, “osteoarthritis”, and “randomized controlled trial”, in conjunction with the Boolean logic operation “OR”/“AND”. Detailed search strategies are provided in Supplementary Tables S1 and S2.

### 2.2. Inclusion/Exclusion Criteria and Study Selection

Using the PICOS framework, included studies focused on Population—patients diagnosed with osteoarthritis; Intervention—probiotics; Comparator—placebo intervention; Outcome—inflammation markers and pain assessment; and Study design—randomized intervention studies.

Reviews, pre-clinical studies, non-randomized trials, cohort or case–control studies, and studies that used probiotics as treatment for other bone diseases were excluded.

**Figure 1:**
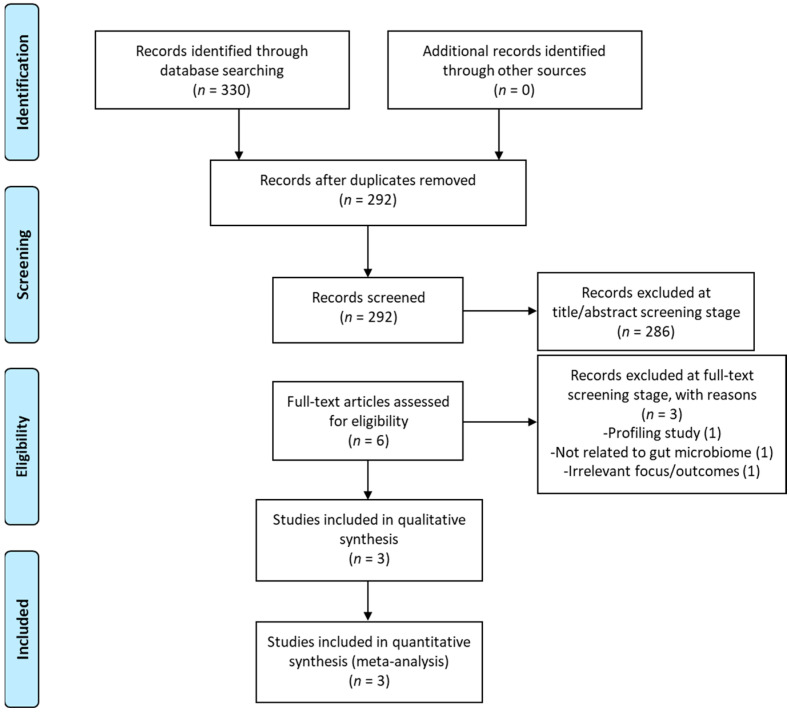
Prisma flow diagram.

Initially, all duplicate studies were identified and removed. Using the predefined inclusion and exclusion criteria, authors M.M. and J. M. performed the title/abstract and full-text reviews. Author A.S. independently verified these two stages of review. In case of a conflict in assessment, an agreement would be sought between the three reviewers (or would be adjudicated by N.F.). The PRISMA flow chart of the included studies strategy is presented graphically in Figure 1.

### 2.3. Outcomes Measures

The measures found to assess the impact of probiotics on OA pain and inflammation in the articles selected for the present systematic review and meta-analysis were the WOMAC (Western Ontario and McMaster Universities Arthritis Index) OA index; patient self-reported pain severity—measured with a 10 cm visual analogue scale (VAS); and serum levels of high-sensitivity C-reactive protein (hs-CRP), a commonly used marker of systemic inflammation.

### 2.4. Data Items and Data Extraction

Data were extracted independently by two reviewers, A.S. and M.M. A third reviewer, N.F., resolved any conflicts. The data extracted included clinical trial report details (authors, year of publication, study design, duration) and patient information (population, age, sample size, gender, treatment groups, intervention administration, and doses). Data for each outcome (WOMAC scores for pain, stiffness, and physical function; VAS pain scores; serum hs-CRP levels), before and after treatment with probiotics or placebo, were also collected.

Mean and standard deviation (SD) or the standard error of the mean (SEM) were extracted from tables or figures using the online tool WebPlotDigitizer (https://apps.automeris.io/wpd4/, accessed on 10 May 2024). For studies that reported mean ± SEM, SD was calculated using the following formula: SEM = SD/sqrt(N). If data on the percent difference from baseline (i.e., before treatment) were provided, they were used directly for the meta-analysis. If instead the measurements of each outcome before and after treatment were provided, then the percent difference was calculated. The SD of the difference between sample means (σ_d_) was calculated using the formula σ_d_ = sqrt(σ_B_^2^/n_1_ + σ_A_^2^/n_2_), where B stands for ‘Before treatment’ and A stands for ‘After treatment’. The standard error (SE) was calculated either from the SD using the formula SE = SD × {sqrt[(1/N_E_) + (1/N_C_)]}, where E stands for ‘Experimental’ and C stands for ‘Control’, or from the t value using the formula SE = MD/t, where MD stands for the ‘difference in means’ [25]. In the absence of variance and SE, the SD was imputed from the weighted average of variances observed in other studies, as previously described [26,27]. In particular, the SD of the WOMAC OA index at week 12 of the Lyu et al., 2020 study [23] was imputed from the weighted average of variances of the WOMAC OA index at 6 months reported in the Lei et al., 2017 study [22].

### 2.5. Data Analysis

Data from the included studies were analyzed from 8 February 2024 to 24 April 2024. Meta-analyses were carried out to identify differences in WOMAC OA index scores, VAS pain scores, and serum hs-CRP levels between the two interventions (probiotics vs. placebo) using the Cochrane Review Manager (RevMan) version 5.3 [28]. The standardized (std.) mean difference was used as the effect measure throughout, either because the unit of measure differed or because different scoring systems were used. Std. mean difference was calculated using the inverse-variance method. A random effect analysis model was used because the data were rather heterogeneous across all outcomes. Additionally, 95% confidence intervals (95% CIs), heterogeneity (I^2^), p-values, and the test for overall effects (*z*-value) were calculated and forest plots were generated. Most analyses were carried out by entering the mean, standard deviation, and number of participants in the probiotics and control groups, apart from the analysis of VAS pain scores, which involved a combined meta-analysis of N-of-1 trial data with randomized controlled trial (RCT) data. In this case, the std. mean difference and SE were entered instead, as previously recommended [29], without indicating the number of participants in each study.

### 2.6. Quality Review

The risk of bias and quality review of the RCTs was conducted by M.M. and J.M. using the revised Cochrane risk-of-bias tool (RoB 2 tool) [30]. This tool assesses and categorizes possible sources of bias arising from the randomization process, deviations from intended interventions, missing outcome data, the measurement of the outcome, and the selection of reported results. Ratings per item were compared and disagreements discussed. If consensus was not reached, A.S. was consulted and the item in question was discussed until consensus was obtained.

The risk of bias assessment for the N-of-1 study was performed by M.M. and A.S. using the Risk of Bias in N-of-1 Trials (RoBiNT) Scale [31]. The scale is a 15-item measure in two subscales: the internal validity (IV) subscale, which reflects the methodological rigor of a study (comprising the items control, randomization, sampling, blinding of investigator, blinding of assessor, interrater agreement, and treatment adherence) and the external validity and interpretation (EVI) subscale, which examines whether the findings of a study can be generalized (comprising the items baseline characteristics, setting, dependent variable, independent variable, raw data report, data analysis, replication, and generalization). Each item is scored using a 3-point scale (0–2) and added together for a total score of 0–30. Summed scores were compared. Any disputes were resolved by discussion or referral to a third reviewer (N.F.).

### 2.7. Certainty of Evidence

The certainty of the evidence was assessed using the grading of recommendations assessment, development, and evaluation (GRADE) approach [32].

### 2.8. Publication Bias

It was not possible to assess publication bias using the funnel plot asymmetry test, because none of the pooled analyses included 10 or more studies, as previously recommended [33]. Therefore, any interpretation should be carried out with a degree of caution.

## 3. Results

### 3.1. Study Selection

A total of 330 records were identified through the database searches and 38 duplicate records were removed. Out of the remaining 292 records, 286 were excluded based on their title and/or abstract. Six full-text records were screened for the inclusion criteria and three of them were excluded. A list of excluded studies, with reasons for their exclusion, is provided in Supplementary Table S3. For the qualitative and quantitative synthesis (meta-analysis), three studies were included, as shown in the PRISMA Flow diagram (Figure 1).

### 3.2. Study Characteristics

Full study characteristics are presented in Table 1. In the Lei et al. study [22], 461 patients with bilateral primary knee OA were randomly assigned into two treatment groups; for a period of 6 months the first group consumed two servings of skimmed milk containing at least 6 × 10^9^ Colony-Forming Units (CFUs) of *Lactobacillus casei* Shirota (LcS) daily (*n* = 230), whilst the second group consumed two servings of plain skimmed milk as a placebo, on a daily basis (*n* = 231). During the 6-month period, 15 patients in the LcS group and 13 patients in the placebo group dropped out, leaving 215 and 218, respectively, in each group.

In the study by Lyu et al. [23], 80 patients with primary knee OA were randomized to the *Streptococcus thermophilus* (TCI633) group (*n* = 41) or the placebo group (*n* = 39). For a period of 12 weeks, the TCI633 group were taking, once daily, four capsules consisting of 5 × 10^8^ bacteria per capsule, whilst the placebo group were taking placebo capsules. No information was provided regarding the composition or the dose of the placebo capsules. Thirteen patients in total dropped out from the study, leaving thirty-seven and thirty patients in the TCI633 group and placebo group, respectively.

The final included study, by Taye et al. [34], reports the results of an N-of-1 trial with two randomized interventions; the active intervention, which included a cocktail of *Lactobacillus rhamnosus*, *Saccharomyces cerevisiae* (*boulardii*), and *Bifidobacterium animalis* ssp lactis, and the placebo intervention. During a period of 32 weeks, the single enrolled participant, a 67-year-old female with OA in her lower back and right ankle, underwent three treatment blocks, each with one pair of active/placebo interventions that were randomly ordered. During each active intervention period, the participant was taking, on a daily basis, two capsules, each containing *Lactobacillus rhamnosus* (LGG^®^) (10 × 10^9^ CFU), *Saccharomyces cerevisiae* (*boulardii*) (7.5 × 10^9^ CFU), and *Bifidobacterium animalis* ssp lactis (BB-12^®^) (5 × 10^9^ CFU), whilst, during each placebo intervention period, the participant was taking, on a daily basis, two matched placebo capsules, each consisting of 400 mg of microcrystalline cellulose. In total there were six intervention periods (three active and three placebo), each lasting 3 weeks and separated by a 2-week washout period.

### 3.3. Meta-Analysis/Forest Plot Interpretation

**Figure 2:**
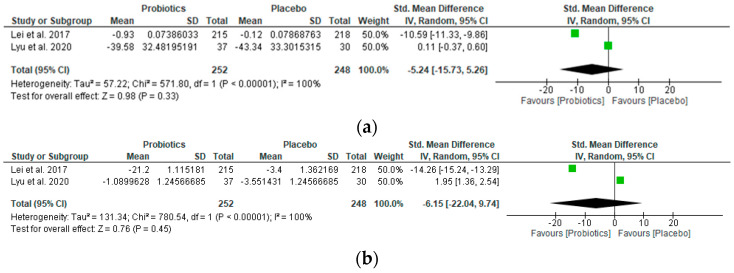
Forest plots showing (**a**) the effect of probiotics on high-sensitivity C-reactive protein (hs-CRP) levels, and (**b**) the effect of probiotics on the WOMAC OA index [22,23]. The green squares represent the estimated std. mean difference for each individual study and the black diamond represents the pooled std. mean difference and the associated 95% CI.

**Figure 3:**
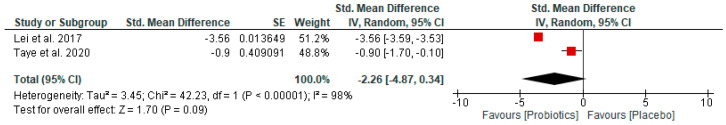
Forest plot showing the effect of probiotics on pain VAS score [22,23]. The red squares represent the std. mean difference for each individual study and the black diamond represents the pooled std. mean difference and the associated 95% CI.

The study outcomes are summarized in forest plots, presenting significant results in favor of probiotic supplementation or placebo for each of the relevant outcomes (Figure 2 and Figure 3). The oral intake of LcS (Lei et al., 2017 [22]; 6-month treatment) and TCI633 (Lyu et al., 2020 [23]; 12-week treatment) did not statistically alter hs-CRP levels in participants (Std. mean difference −5.24 [95% CI −15.73, 5.26]; *z*-value 0.98; *p*-value 0.33) (Figure 2a). Similarly, the oral intake of these probiotics did not decrease the WOMAC OA index score in participants (Std. mean difference −6.15 [95% CI −22.04, 9.74]; *z*-value 0.76; *p*-value 0.45) (Figure 2b), neither did it significantly lower the WOMAC pain, stiffness, or physical function subscale scores (Table 2).

It is interesting to observe that, with the exception of the stiffness score, the probiotic strain LcS used in Lei et al.’s RCT consistently provided a significant reduction in symptoms across all outcomes measured, whilst the opposite was observed for Lyu et al.’s study with TCI633, which consistently performed as good as or worse than the placebo across all outcomes except stiffness.

As for Taye et al.’s use of a cocktail of *Lactobacillus rhamnosus*, *Saccharomyces cerevisiae* (*boulardii*), and *Bifidobacterium animalis* (Taye et al., 2020 [34]; 3-week treatment), this did not perform any better than the placebo on the only measured outcome of perceived pain. However, the pooled intervention effect estimate with the oral intake of LcS (Lei et al., 2017 [22]; 6-month treatment) suggests a moderate reduction in the osteoarthritis pain VAS score (Std. mean difference −2.26 [95% CI −4.87, 0.34]; *z*-value 1.70; *p*-value 0.09) (Figure 3).

### 3.4. Risk of Bias of Included Studies

**Figure 4:**
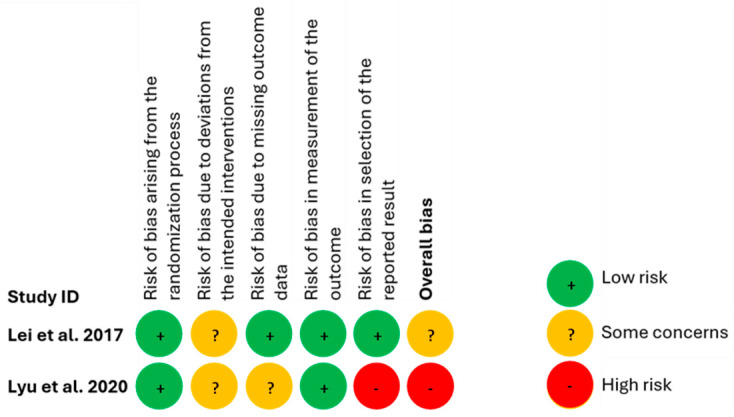
Risk of bias for included RCT studies [22,23].

Findings from the evaluation of the risk of bias, using the RoB 2 tool, for the RCTs are presented in Figure 4. Lei et al., 2017 [22] study raised some concerns because deviations from the intended interventions were detected. Lyu et al. (2020) [23], however, were deemed to be at a high risk of bias primarily because an important risk of bias was identified in the selection of their reported results.

The findings from the evaluation of the risk of bias with the RoBiNT scale for the N-of-1 trial are shown in Table 3. On a 0–14 scale, its internal validity was 10, while its external validity and interpretation was 11 on a scale of 0–16. Its total score was 21 out of 30. Items 7 (treatment adherence) and 14 (replication) scored 0. The second lowest scoring items, which scored 1, were items 5 (blinding of assessors) and 6 (interrater agreement) from the internal validity subscale and items 8 (baseline characteristics), 9 (setting), and 12 (raw data record) from the external validity and interpretation subscale. An algorithm used to evaluate the methodological rigor of the internal validity of the study [35] showed that the N-of-1 study included in this systematic review [34] was found to be fair, suggesting that there may be a substantial risk of bias.

### 3.5. Certainty of Evidence

The effect of probiotics on the outcomes assessed (hs-CRP levels, VAS pain scores, WOMAC OA index) was rather uncertain due to the high risk of bias in one of the studies; study design heterogeneity, suggesting inconsistency; and wide confidence intervals, indicating moderate imprecision. Given these reasons, the certainty of the evidence was found to be very low.

## 4. Discussion

This review and meta-analysis present an investigation into the preliminary evidence of the potential effect of probiotics on improving the status of OA-related factors such as pain and inflammation markers. Data are synthesized from three randomized control trials (one being a single N RCT) including 501 human participants. The results from the meta-analysis suggest that probiotics might be of use in the treatment of OA in humans. Although the specific mechanism through which probiotics affect OA outcomes is still under investigation, several preclinical and clinical studies [6] suggest that probiotics affect the gut microenvironment by modulating the activity of certain gut microbiota and reducing the release of pro-inflammatory cytokines (e.g., TNF-α, IL-7), which then leads to a lower chronic inflammatory response that underlies OA characteristics such as the inflammation of the synovial membrane [7]. The reviewed studies did not assess these potential mechanistic pathways as part of the effect of probiotics in OA; therefore, more complex studies that allow for these processes to be investigated are needed.

The findings from the meta-analysis were mixed, with a clear differential effect of probiotics depending on the strain used in each study. Overall, this article suggests a potential beneficial effect of the *Lactobacillus* strain of probiotics vs. the *Streptococcus* strain across all outcomes, except for self-reported joint stiffness. This finding seems to be in line with some clinical data that demonstrate that *Lactobacillus* leads to a decrease in various inflammatory factors as well as nociceptive mediators and is generally correlated to a decrease in OA symptoms [36,37]. Conversely, the presence of higher concentrations of bacterial strains such as *Streptococcus* in the gut have been linked to higher levels of pain and disability in OA patients. Similarly, the *Lactobacillus* strain seems to be a promising treatment intervention for other types of arthritis such as rheumatoid arthritis (RA), with a recent review and meta-analysis highlighting the potential of this strain in reducing CRP [38]. The impact of *Lactobacilli* in OA had already been preliminarily stated in a previous systematic review and meta-analysis [39]; however, this previous article had identified only one RCT [22]. Our current effort adds to the literature by further contextualizing the potential of *Lactobacillus* probiotics in OA when compared to alternative probiotic supplementation [23,24]. Preclinical evidence also echoes these findings. The systematic review by Marchese et al. (2023) showed that *Lactobacilli* in particular have been methodically studied over the years in rodent OA models and shown to effectively prevent cartilage damage, reduce inflammation, and alleviate OA-related pain in monotherapy, as well as in combination with other effective OA treatments [6]. In view of these results, and based on the current evidence, it seems that *Lactobacillus* might be a more promising strain in the treatment of OA symptoms; however, more studies are needed.

Interestingly, as nutraceuticals, probiotics are not unique in addressing the triad of common OA symptoms. Other nutraceuticals have recently been shown to alleviate OA symptoms, including pain and joint function. Farì and colleagues (2023) showed that treatment with hemp seed oil combined with terpenes is more effective in relieving knee OA pain, improving knee function, and reducing knee OA inflammation than treatment with hemp seed oil alone [40,41]. The authors therefore suggested that hemp seed oil and terpenes could be considered a good complementary option for OA-suffering patients.

There are several limitations to this review and meta-analysis, the main one being the very low number of studies synthesized and the low N reported (only three, with a combined N of 501 participants). This is reflective of the infancy of human trials on the effect of probiotics on OA, which is confirmed by the low number of registered trials on this subject on ClinicalTrials.gov (currently only one trial; TCI633). Regarding the Ns of the reviewed trials, one trial [22] seemed to be at least adequately powered, with a N of 433; however, there are significant power issues with the remaining studies. There are also some concerns with the risk of bias presented in the reviewed studies, with the best of them being at moderate risk of bias. Given the small number of studies, the presence of bias can significantly skew the findings. Therefore, careful consideration needs to be given to the evidence presented. Finally, there were issues with heterogeneity across studies, with different strains of probiotics being used across the three trials, different methodologies being used to assess the effect (RCT vs. N-of-1 RCT), different measurements of the outcomes, and some outcomes being measured in a self-report format rather than using objective assessments. However, this review and meta-analysis is the first of its kind and proposes a set of guiding principles in relation to future trials to better ascertain the effectiveness of the use of probiotics in OA treatments.

The first essential component would be a form of process measurement based on microbiome richness and diversity. One possibility would be the use of 16S rRNA gene sequencing for the species- and strain-level microbiome at pre-, mid-, and post-treatment time points [42]. This would ensure more homogeneity in sample selection, or the possibility to adjust for heterogeneity in these parameters at later stages of statistical analyses for the clinical significance of treatment (e.g., interactions between microbiome changes and clinical outcome changes).

A second point would be to control for diet within the trial period. This might involve, for example, providing a list of foods or ingredients that the participants should avoid or minimize their consumption of in order to ensure that additional strains of probiotics are not causing confounding effects. The dietary prescription described in the protocol by Jansson et al. [43] would be a good start.

Thirdly, there should be an incorporation of more directly measurable outcomes beyond inflammatory markers (e.g., CRP). An example would be the assessment of OA-related changes in cartilage via MRI-based cartilage morphometry, a non-invasive method for the assessment of articular cartilage (reviewed in [44]).

Finally, for self-reported outcomes, future studies should consider more reliable alternatives to the traditional average of experience over a period of time (e.g., how many days in pain over the last week). This is essential as outcomes such as pain, stiffness, and functional impairment are highly susceptible to environmental and contextual features. For example, a person’s perception of pain varies widely depending on their mood, time of day, and what activity they might be engaged in [45]. The introduction of measurement models such as the ecological momentary assessment (EMA) model would improve the reliability of these outcomes greatly. Stone and colleagues provide a comprehensive guide on the use of EMA for pain from the conceptual level to its implementation in clinical trials [46,47,48].

## 5. Conclusions

In this review, we have comprehensively examined the evidence of the use of probiotics in treating the OA patient symptoms of pain and inflammation. The results are mainly inconclusive due to the scarcity and heterogeneity of these studies; however, the evidence, coupled with preclinical and clinical studies, suggests that *Lactobacillus* strains of probiotics might be of use. We propose a pathway for the continuation of this line of research that will reduce some of the bias in the evidence.

## Figures and Tables

**Table 1:** Characteristics of included studies.

Study/Year	Population	Age (Years)	No. of Patients after Dropout (No. Male/Female)	Study Design	Treatment Groups (*n*)	Treatment Dose	Treatment Period	Outcomes Reported ^1^
Lei, et al., (2017) [22]	Patients with knee OA	66.9 ± 5.0	433 (192/241)	RCT	LcS (*n* = 215) Placebo (*n* = 218)	Two servings of skimmed milk containing at least 6 × 10^9^ CFU of *Lactobacillus casei* Shirota, daily Two servings of plain skimmed milk, daily	6 months	***WOMAC score*** ***VAS pain score*** ***Serum hs-CRP***
Lyu, et al., (2020) [23]	Patients with knee OA	60.8 ± 12.2	67 (14/53)	RCT	TCI633 (*n* = 37) Placebo (*n* = 30)	Four TCI633 capsules containing 5 × 10^8^ bacteria per capsule, daily Placebo capsules, daily	12 weeks	K/L grade ***WOMAC score*** ***Serum hs-CRP*** Serum CTX-II
Taye, et al., (2020) [34]	Patients with OA in lower back and right ankle	67	1 (0/1)	N-of-1 trial	Active intervention: capsule of *Lactobacillus rhamnosus* (LGG^®^), *Saccharomyces cerevisiae (boulardii)*, and *Bifidobacterium animalis ssp lactis* (BB-12^®^) Placebo intervention: matched capsule with microcrystalline cellulose	Active intervention: Two capsules containing *Lactobacillus rhamnosus* (LGG^®^) (10 × 10^9^ CFU), *Saccharomyces cerevisiae (boulardii)* (7.5 × 10^9^ CFU), and *Bifidobacterium animalis ssp lactis* (BB-12^®^) (5 × 10^9^ CFU), daily Placebo intervention: Two capsules containing 400 mg of microcrystalline cellulose, daily NOTE: Three treatment blocks, each with one pair of active/placebo interventions, randomly ordered	Six intervention periods, each lasting 3 weeks and separated by a 2-week washout period In total: 32 weeks	***VAS pain score*** Patient-Specific Functional Scale (PSFS) Participant Preference Comprehensive Digestive Stool Analysis (CDSA) Rescue Medication Usage General Health Questionnaire (GHQ-12)

^1^ Outcomes used for meta-analysis are shown in ***bold and italics***. OA, osteoarthritis; RCT, randomized controlled trial; LcS, *Lactobacillus casei* Shirota; TCI633, *Streptococcus thermophilus*; CFU, colony forming unit.

**Table 2:** Summary of meta-analysis results for the WOMAC pain, stiffness, and physical function subscale scores.

Outcome	Std. Mean Difference (95% CI)	Statistical Method	Test for Heterogeneity	Test for Overall Effect
WOMAC pain subscale score	−1.13 (−13.32, 11.05)	Std. mean difference, IV, Random effects, 95% CI	Chi^2^ = 457.60, *df* = 1, *p* < 0.00001, *I*^2^ = 100%	*z* = 0.18, *p* = 0.86
WOMAC stiffness subscale score	−21.31 (−63.41, 20.79)	Std. mean difference, IV, Random effects, 95% CI	Chi^2^ = 125.52, *df* = 1, *p* < 0.00001, *I*^2^ = 99%	*z* = 0.99, *p* = 0.32
WOMAC physical function subscale score	−4.59 (−20.75, 11.56)	Std. mean difference, IV, Random effects, 95% CI	Chi^2^ = 740.16, *df* = 1, *p* < 0.00001, *I*^2^ = 100%	*z* = 0.56, *p* = 0.58

**Table 3:** Evaluation of the risk of bias for the N-of-1 study using the RoBiNT scale.

Study ID	Internal Validity (IV) Subscale ^1^							Total IV (Out of 14)	External Validity and Interpretation (EVI) Subscale ^2^								Total EVI (Out of 16)	Total Score (Out of 30)
	1	2	3	4	5	6	7		8	9	10	11	12	13	14	15		
Taye et al., 2020 [34]	2	2	2	2	1	1	0	10	1	1	2	2	1	2	0	2	11	21

^1^ Internal validity (IV) subscale: 1, design with control; 2, randomization; 3, sampling of behavior; 4, blinding of people involved in the intervention; 5, blinding of assessor(s); 6, interrater agreement; 7, treatment adherence. ^2^ External validity and interpretation (EVI) subscale: 8, baseline characteristics; 9, setting; 10, dependent variable (target behavior); 11, independent variable (therapy/intervention); 12, raw data record; 13, data analysis; 14, replication; 15, generalization.

## Supplementary Materials

The following supporting information can be downloaded at https://www.mdpi.com/article/10.3390/nu16142243/s1, Table S1. Search strategy and keywords for Medline and Embase; Table S2. Search strategy and keywords for Web of Science; Table S3. Details of studies excluded at full-text screening stage; Table S4. PRISMA 2020 Checklist; Table S5. PRISMA 2020 for Abstract Checklist. References [49,50,51] are cited in Supplementary Materials.
